# No association between genetically predicted vitamin D levels and Parkinson’s disease

**DOI:** 10.1371/journal.pone.0313631

**Published:** 2024-11-15

**Authors:** Zihao Wang, Huan Xia, Yunfa Ding, Ranran Lu, Xinling Yang

**Affiliations:** 1 Department of Neurology, The Second Affiliated Hospital of Xinjiang Medical University, Xinjiang, China; 2 Jinshazhou Hospital of Guangzhou University of Chinese Medicine, Guangzhou, China; Fondazione Don Carlo Gnocchi, ITALY

## Abstract

**Background:**

Parkinson’s disease (PD) is a neurodegenerative disorder, primarily characterized by motor impairments. Vitamin D has several regulatory functions in nerve cell survival and gene expression via its receptors. Although research has shown that vitamin D deficiency is prevalent among PD patients, the causal link to PD risk remains unclear. This study aims to investigate the causal relationship between vitamin D and PD using a bidirectional two-sample Mendelian randomization (MR) analysis method.

**Methods:**

This study applied a bidirectional two-sample MR analysis to explore the causal link between vitamin D and PD. We selected statistically significant single nucleotide polymorphisms (SNPs) related to 25-hydroxyvitamin D (25(OH)D) as instrumental variables (IVs), ensuring no association with known confounders. The analysis used GWAS data from over 1.2 million Europeans across four major published datasets, elucidating the genetic correlation between vitamin D levels and PD.

**Results:**

We identified 148 instrumental SNPs associated with 25(OH)D. After adjustment for confounding-related SNPs, 131 SNPs remained in the analysis. Data from three PD cohorts revealed no significant correlation between 25(OH)D levels and PD risk using the IVW method (*P*_cohort1_ = 0.365, *P*_cohort2_ = 0.525, *P*_cohort3_ = 0.117). The reverse MR analysis indicated insufficient evidence of PD causing decreased vitamin D levels (*P* = 0.776).

**Conclusion:**

This is the first study to use bidirectional MR across three PD cohorts to investigate the causal relationship between vitamin D and PD. The results indicate that vitamin D levels are not significantly causally related to PD risk at the genetic level. Therefore, future studies should exercise caution when investigating the relationship between vitamin D levels and PD risk. While no direct causal link exists between vitamin D levels and PD, this does not preclude the potential of vitamin D levels as a biomarker for PD diagnosis. Furthermore, larger-scale longitudinal studies are necessary to evaluate the diagnostic and predictive value of vitamin D levels in PD.

## 1 Introduction

Parkinson’s disease (PD) is a complex neurodegenerative disorder involving both genetic and environmental factors [1, 2]. The primary symptoms of PD are resting tremor, muscle rigidity, bradykinesia, and postural instability [3]. PD patients also may experience non-motor symptoms including cognitive impairments, emotional issues, and sleep disturbances [4]. Research shows that low serum levels of vitamin D correlate with psychiatric disorders such as depression, bipolar disorder, schizophrenia, and neurodegenerative diseases including dementia and PD [5]. Vitamin D receptors are present in neurons and glial cells throughout key brain regions, such as the substantia nigra, hippocampus, hypothalamus, thalamus, and basal ganglia. In these areas, vitamin D influences neuronal differentiation and maturation, regulates the synthesis of growth factors, and supports the production of neurotransmitters like acetylcholine, dopamine, and gamma-aminobutyric acid [6, 7]. Vitamin D, available in D2 and D3 forms, is now considered a hormone involved in multiple regulatory mechanisms essential for neuronal survival [8]. As a fat-soluble steroid, vitamin D is essential for bone metabolism, regulates the calcium and phosphate balance, and modulates the expression of many genes via its receptor [9]. Vitamin D metabolites are able to cross the blood-brain barrier, and 1,25-dihydroxyvitamin D3 in cerebrospinal fluid signifies active vitamin D metabolism in the central nervous system [10]. Under UVB radiation, vitamin D3 is synthesized in the skin from 7-dehydrocholesterol and is subsequently converted to 25-hydroxyvitamin D (25(OH)D) in the liver, and finally to 1,25-dihydroxyvitamin D in the kidneys [11]. Serum levels of 25(OH)D are routinely measured to assess vitamin D status.

Studies reveal that vitamin D deficiency is significantly more severe among PD patients compared to the general population [12]. Considering the neuroprotective role of vitamin D, it may function by promoting neural growth or inhibiting cytotoxicity [13]. Mehanna and colleagues observed that serum levels of 25(OH)D and total 25(OH)D are lower in PD patients than in control groups [14]. However, findings on the relationship between vitamin D and non-motor symptoms in PD remain inconsistent. Shrestha and colleagues found no significant association between serum 25(OH)D levels and the risk of PD in a prospective study [15]. Data from vitamin D intake studies suggest that prolonged vitamin D deficiency does not impair the integrity of the dopamine system, although these findings are not entirely consistent with other research [16].

Mendelian randomization (MR) is a novel genetic epidemiological method that uses genetic variants closely associated with specific exposures as IVs, effectively reducing confounders and enhancing the accuracy of causal inference [17]. Currently, no studies have explored the causal relationship between vitamin D genetic variants and PD risk, nor have any evaluated the impact of PD on vitamin D levels. Our study aims to explore the causal connection between vitamin D and PD risk using a bidirectional two-sample MR analysis.

## 2 Materials and methods

### 2.1 Study design and data source

To explore the potential causal link between 25(OH)D levels and PD, we conducted a two-sample MR analysis. The validity of instrumental variables (IVs) hinges on three critical assumptions. First, the genetic variants used as IVs should be significantly associated with the exposure factor (25(OH)D). Second, these genetic variants must not correlate with any confounding factors. Third, the genetic variants should influence the outcome (PD) solely through the exposure, without alternative pathways.

The 25(OH)D GWAS data originate from a genome-wide association study by Manousaki et al. in the UK Biobank cohort, involving SNP data from 401,460 individuals of European ancestry. The PD data are sourced from three PD outcome cohorts’ GWAS data (GWAS IDs: "ieu-b-7", "ieu-a-812", and "finn-b-G6_PARKINSON"). The first PD outcome cohort comes from the latest genome-wide association study (GWAS) conducted by the International PD Genomics Consortium (IPDGC), which included three previously reported GWAS studies, 13 new datasets, and proxy case data from the UK Biobank (33,674 cases and 449,056 controls). The second PD outcome cohort includes 1,713 Caucasian patients and 3,978 healthy controls. The third PD outcome cohort, we used the GWAS dataset from the FinnGen Consortium for MR analysis (4,681 cases and 407,500 controls). We selected GWAS data from European ancestry individuals mainly because of high-quality genetic data available in this population. These datasets encompass broad genetic variation, enhancing the robustness of MR analysis. The Finnish GWAS dataset was incorporated to utilize its large sample size and rich genetic data, which complement other European datasets. Despite Finland’s relatively isolated population and unique genetic characteristics, its overall genetic structure closely mirrors that of broader European populations, minimizing the potential impact of population stratification on the results.

**Fig 1:**
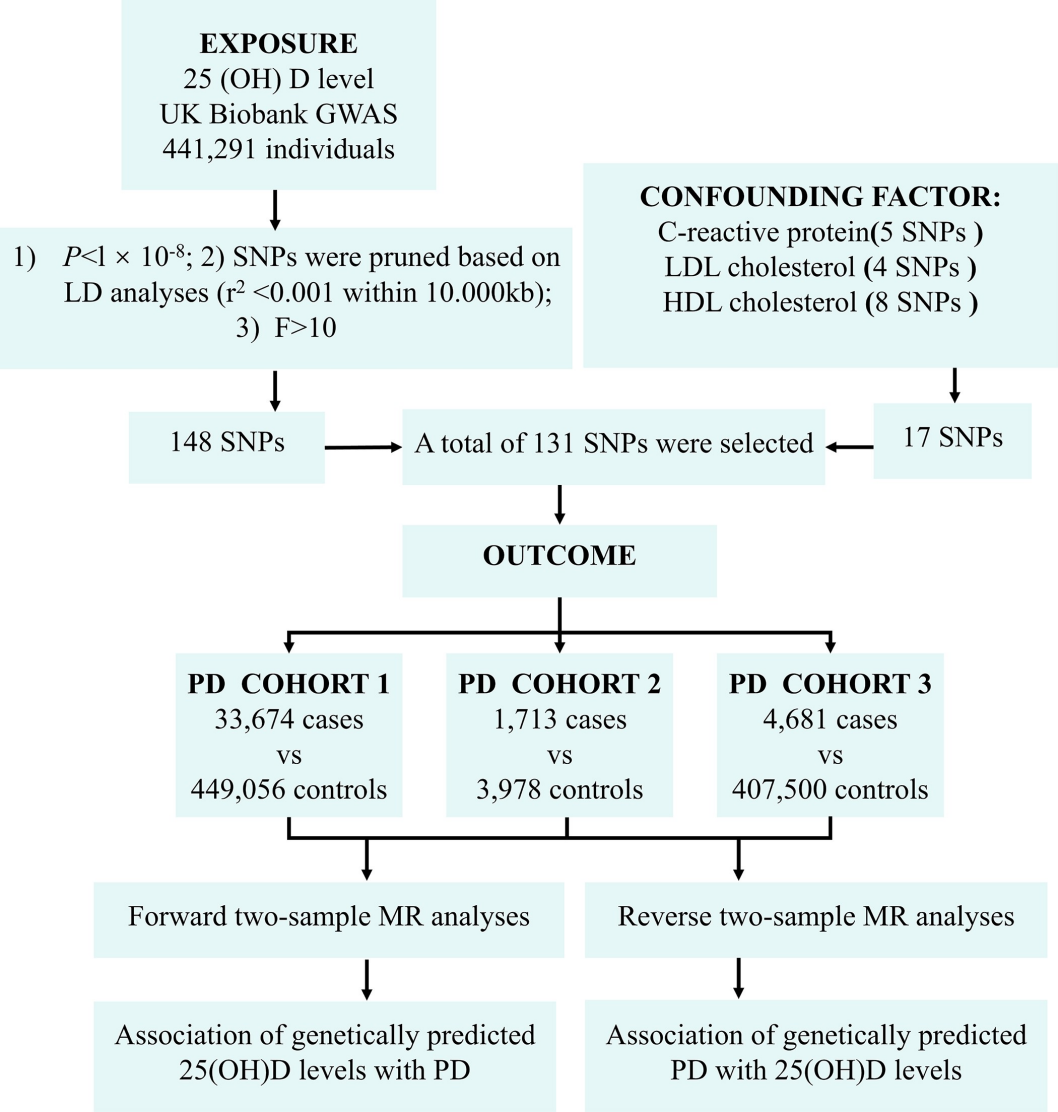
Workflow for selecting IV and MR analysis.

The data used in this study are publicly accessible and have been officially approved by the Medical Ethics Committee of the Second Affiliated Hospital of Xinjiang Medical University (Grant No. 2022K004). The study design and implementation steps are shown in Fig 1.

### 2.2 Screening genetic IVs

To satisfy the first assumption of MR analysis, that IVs are strongly associated with 25(OH)D, we selected independent IVs that are statistically significantly associated with 25(OH)D across the whole genome (*P* < 5 × 10^−8^, r^2^ < 0.001, genetic distance = 10,000 KB, minor allele frequency > 0.01). We selected single nucleotide polymorphisms (SNPs) with an F-value greater than 10 to secure the association’s stability and minimize bias from weak IVs. To avoid potential confounding effects of genetic variations, we consulted the PhenoScanner database to verify the association of selected IVs with any established confounding factors [18]. Currently known risk factors for PD include C-reactive protein, LDL cholesterol, and HDL cholesterol. Hence, this study excluded SNPs whose genetic variations are related to these factors.

### 2.3 Statistical analysis

We compared the aggregate statistics of the selected IVs with datasets from three PD cohorts across different GWAS. Five MR analysis were used, among which the inverse-variance weighted (IVW) method has the highest statistical power and is typically used when all IVs are valid. Other MR analyses included the weighted median method, MR-Egger method, weighted mode, and simple mode. The heterogeneity of SNPs estimates was assessed using Cochrane’s Q test, and MR-PRESSO analysis was used to identify and correct potential pleiotropic outlier effects. By systematically excluding each SNP, it was determined whether any individual SNP significantly influenced the estimates. Statistical analyses were performed using the R packages mendelianrandomization, MRPRESSO, and TwoSampleMR in R version 4.1.2.

## 3 Results

### 3.1 Association of genetically predicted vitamin D levels with PD

**Fig 2:**
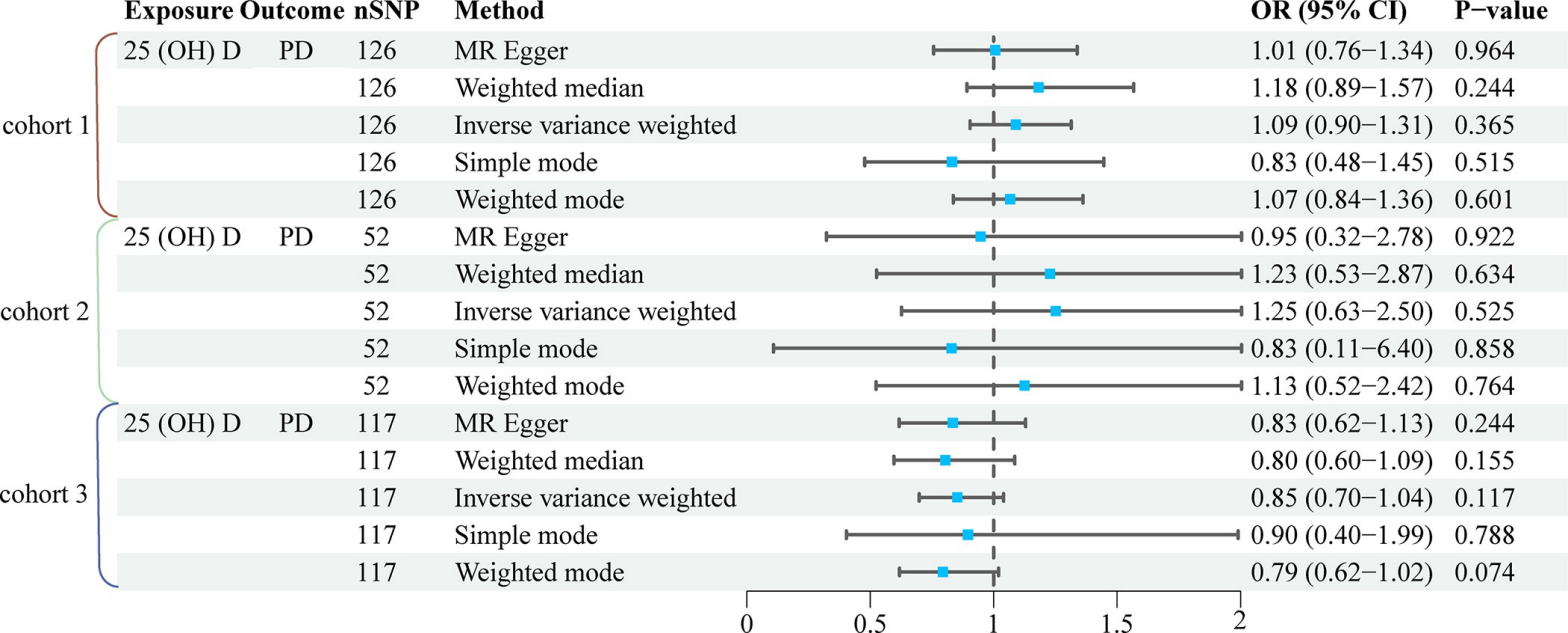
MR analysis of the relationship between 25 (OH) D and the risk of PD. CI, confidence interval; OR, odds ratio; nSNP, number of single nucleotide polymorphism.

To explore the genetic association between vitamin D levels and PD, we first selected independent SNPs associated with serum 25(OH)D levels from GWAS datasets. The selection criteria for these SNPs included: p < 5 × 10⁻⁸, r^2^ < 0.001, a genetic distance of 10,000 KB, and a minor allele frequency greater than 0.01. Subsequently, we further filtered SNPs with an F-statistic greater than 10, ultimately identifying 148 SNPs related to 25(OH)D, which were used as IVs. The F-statistics related to the genetic instruments for 25(OH)D ranged from 25.33 to 2448.32, with an average of 106.64. After excluding SNPs associated with C-reactive protein (rs79598313, rs7528419, rs1229984, rs7314285, rs58542926), LDL cholesterol (rs11127048, rs964184, rs1883711, rs960596), and HDL cholesterol (rs10864726, rs1047891, rs7828742, rs532436, rs7910135, rs1800588, rs1800775, rs4121823), 131 SNPs related to 25(OH)D were included for analyzing the association with PD (S1 Table). In three PD cohorts, the IVW method showed no significant statistical relationship between 25(OH)D levels and PD (*P*_cohort1_ = 0.365, *P*_cohort2_ = 0.525, *P*_cohort3_ = 0.117). Other methods such as the weighted median, simple mode, and weighted mode provided similar results (Fig 2). Apart from a significant heterogeneity suggested in the causal relationship between 25(OH)D and PD cohort 2 (P<0.05), Cochran’s Q test, MR-Egger regression, and leave-one-out analysis showed no notable heterogeneity or pleiotropy (Table 1 and S1 Fig).

**Table 1:** Pleiotropy and heterogeneity tests of forward MR analysis.

Test	Method	Cohort	*P*
Heterogeneity	Q MR Egger	1	0.111
	Q IVW	1	0.117
Pleiotropy	MR-Egger regression	1	0.468
Heterogeneity	Q MR Egger	2	0.019
	Q IVW	2	0.021
Pleiotropy	MR-Egger regression	2	0.468
Heterogeneity	Q MR Egger	3	0.250
	Q IVW	3	0.270
Pleiotropy	MR-Egger regression	3	0.857

### 3.2 Association of genetically predicted PD with vitamin D levels

**Fig 3:**
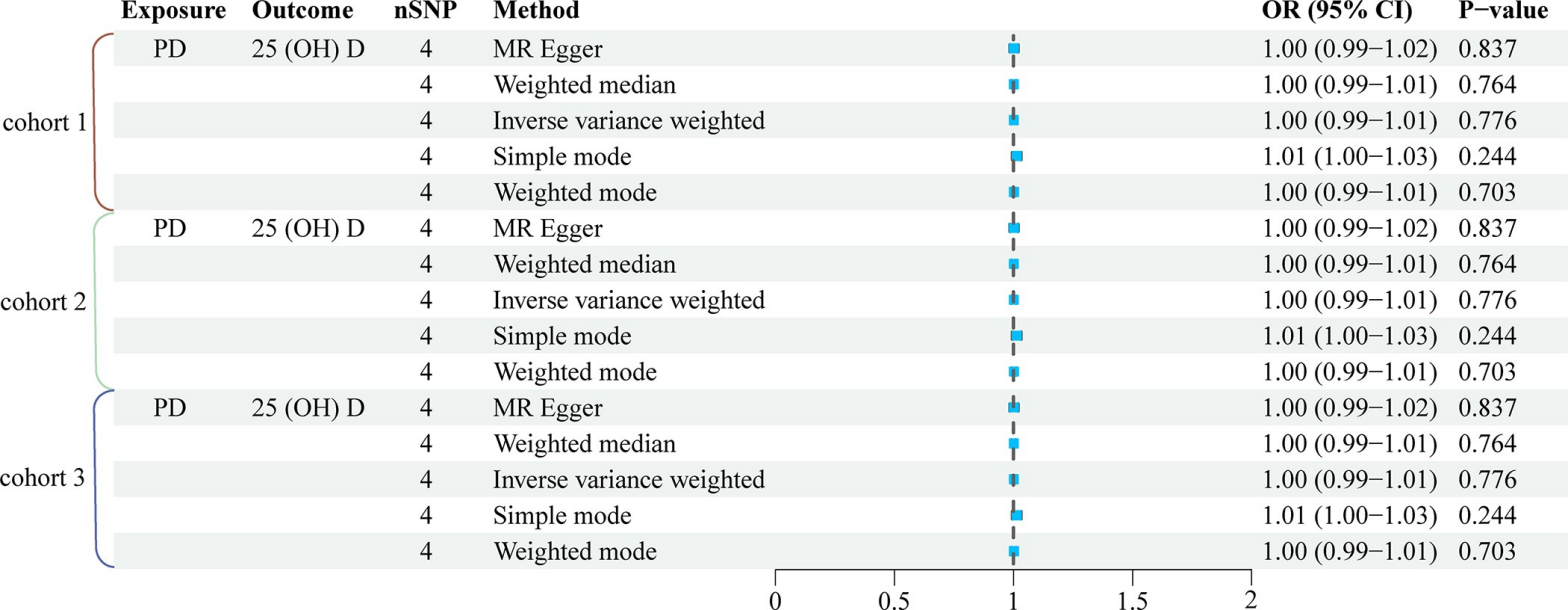
MR analysis of the relationship between PD and the risk of 25 (OH) D.

Reverse MR analysis was conducted to examine the association between genetically predicted PD and vitamin D levels. Three PD cohorts shared the same 4 SNPs (rs35603727, rs2583990, rs117503845, rs2732613). Our findings indicate that PD does not influence 25(OH)D levels. No statistically significant association between PD and vitamin D levels was observed in any of the cohorts (P > 0.05). This suggests that while a potential inverse relationship may exist, it is not strong enough to reach statistical significance within our dataset. Other analytical approaches, including weighted median, simple mode, and weighted mode, also showed results analogous to the IVW method, as illustrated in Fig 3. Moreover, tests including Cochran’s Q, MR-Egger regression, and leave-one-out analysis provided no substantial evidence of heterogeneity or pleiotropy (Table 2 and S2 Fig).

**Table 2:** Pleiotropy and heterogeneity tests of reverse MR analysis.

Test	Method	Cohort	*P*
Heterogeneity	Q MR Egger	1, 2, 3	0.085
	Q IVW	1, 2, 3	0.174
Pleiotropy	MR-Egger regression	1, 2, 3	0.932

## 4 Discussion

As the global population grows and life expectancy increases, the incidence of neurological diseases rises as well [19, 20]. PD mechanisms include inflammation [21], oxidative stress [22], mitochondrial dysfunction [23], lysosomal defects [24], impaired RNA homeostasis [25], and the misfolding and aggregation of certain proteins (such as α-synuclein, amyloid-beta, and hyperphosphorylated tau) [26]. Low serum vitamin D levels are associated with various mental illnesses (such as depression, bipolar disorder, schizophrenia) and neurodegenerative diseases (such as dementia and PD). Research indicates a significant association between lower serum vitamin D levels and an increased risk of developing PD. For instance, Knekt and colleagues [27] conducted a large prospective cohort study, revealing that individuals with lower serum 25(OH)D levels had a higher incidence of PD over a 29-year follow-up period. The meta-analysis results regarding the association between vitamin D receptor (VDR) polymorphisms and PD risk are inconsistent. Wang et al. [28] indicated that SNP FokI is associated with a reduced PD risk in Asian populations, but not in Caucasians. Although rs2228570 shows a significant association with PD risk in some models, particularly in Asian populations, the results for rs731236, rs7975232, and rs1544410 do not consistently show a significant association with PD risk. Studies reveal vitamin D receptors on dopaminergic neurons in the human substantia nigra, suggesting that vitamin D may protect these neurons [29]. Although not yet directly confirmed in humans, studies in animal models suggest that vitamin D may have a protective effect on dopaminergic neurons [30]. However, the evidence for vitamin D’s role in PD pathogenesis is still insufficient. A comprehensive cohort study indicates no direct association between long-term vitamin D intake and the integrity of the dopamine system, thus suggesting no link with PD risk [16]. Moreover, Shrestha and colleagues’ prospective study found no significant link between serum 25(OH)D levels and PD risk [15], aligning with the findings of this study’s forward MR analysis. Osteoporosis, particularly prevalent in women with PD [31], is linked to low bone density and calcium levels, increasing hip fracture risk [32]. Studies indicate significantly lower vitamin D levels in PD patients compared to healthy controls [33–35]. Research attributes reduced vitamin D synthesis in PD patients to decreased mobility and prolonged disease progression, which limits sunlight exposure [36]. Marian and colleagues [37] discovered that patients with early-stage PD commonly exhibit vitamin D deficiency. Moreover, the vitamin D levels remained stable without any decline as the disease progressed. Thomas’ study did not identify a significant association between vitamin D levels and clinical parameters, including disease severity scores or cognitive function [38]. This finding suggests that vitamin D levels may not be a reliable marker for the progression of PD. The potential benefits of vitamin D supplementation in patients with PD remain unclear, and the study does not offer conclusive evidence to support or oppose its use. Another study shows a slight but insignificant difference in serum vitamin D concentrations between PD patients and control groups [39]. This finding aligns with the outcomes from our reverse MR analysis.

It should be noted that the aforementioned studies are observational. Observational studies are commonly used to explore relationships between phenotypes and diseases but cannot establish causality. To overcome this limitation, we utilized MR analysis to investigate the potential causal relationship between vitamin D levels and PD. Currently, no causal studies have assessed the impact of PD on vitamin D levels. Bidirectional MR analysis using two samples revealed no significant association between vitamin D levels and PD. The possible reasons may attribute to the following. First, the pathological changes in PD are closely related to the aggregation of misfolded α-synuclein, and changes in vitamin D levels may not accurately reflect the actual state of the brain. Second, although vitamin D levels are lower in PD patients, this deficiency does not directly cause PD. Third, reduced activity in PD patients may lead to insufficient vitamin D intake, suggesting that the decrease in vitamin D levels is a result of PD rather than a cause. Fourth, vitamin D might impact PD progression through its immune modulation and anti-inflammatory effects, which may not be fully reflected in our genetic analysis. Additionally, vitamin D could influence PD via calcium metabolism or neuroprotective mechanisms. Some studies have failed to adequately control for confounding factors such as sun exposure, dietary habits, and body mass index, which may have led to inconsistent results. Therefore, caution should be exercised when exploring the relationship between vitamin D levels and PD risk in the future. Although there is no direct causal relationship between vitamin D levels and PD, this does not exclude the potential of vitamin D levels as a biomarker for PD diagnosis. Additionally, larger-scale longitudinal studies are needed in the future to assess the diagnostic and predictive value of vitamin D levels in PD.

Our study boasts multiple advantages. It is the first to evaluate the association between vitamin D and PD through bidirectional MR analysis, confirmed by three separate PD cohorts for reliability. MR analysis employs SNPs as IVs to evaluate causal relationships between exposure factors and outcomes. Secondly, the study integrates data from over 1.2 million Europeans across four widely-published GWAS datasets, improving our understanding of the genetic links between vitamin D levels and PD, and minimizing the effects of population stratification. Thirdly, we excluded known confounders like C-reactive protein [40–42], LDL cholesterol, and HDL cholesterol [43–45] to ensure clarity in our findings. However, our MR analysis has limitations that need consideration. Although some studies deem a single 25(OH)D measurement reliable for assessing vitamin D levels, baseline serum 25(OH)D measurements might not reflect long-term levels [46]. Additionally, as the study sample comprises individuals of European descent, the findings may not generalize to other ethnicities, underscoring the need for further research to validate these results. Lastly, although we have adjusted for potential confounding factors as much as possible, residual or unmeasured confounding factors cannot be completely excluded.

## 5 Conclusion

This is the first study to use bidirectional MR across three PD cohorts to investigate the causal relationship between vitamin D and PD. The results indicate that vitamin D levels are not significantly causally related to PD risk at the genetic level. Therefore, future studies should exercise caution when investigating the relationship between vitamin D levels and PD risk. While no direct causal link exists between vitamin D levels and PD, this does not preclude the potential of vitamin D levels as a biomarker for PD diagnosis. Furthermore, larger-scale longitudinal studies are necessary to evaluate the diagnostic and predictive value of vitamin D levels in PD.

## Supporting information

S1 Checklist STROBE-MR checklist of recommended items to address in reports of Mendelian randomization studies. (DOCX)

S1 Table SNPs associated with 25(OH)D. (DOCX)

S1 Fig Leave-one-out analyses for each SNP-25(OH)D association. (DOCX)

S2 Fig Leave-one-out analyses for each SNP-PD association. (DOCX)
