# Supplementary material for: No association between genetically predicted vitamin D levels and Parkinson’s disease
Source: PLoS One. 2024 Nov 15;19(11):e0313631. doi: 10.1371/journal.pone.0313631 (PMC11567546; doi:10.1371/journal.pone.0313631)
Supplement: S1 Checklist — (DOCX) [file pone.0313631.s001.docx]

**STROBE-MR checklist of recommended items to address in reports of Mendelian randomization studies**^1^ ^2^

| **Item No.** | **Section** | **Checklist item** | **Page No.** | **Relevant text from manuscript** |
| --- | --- | --- | --- | --- |
| 1 | **TITLE and ABSTRACT** | Indicate Mendelian randomization (MR) as the study’s design in the title and/or the abstract if that is a main purpose of the study | 1 | No association between genetically predicted Vitamin D levels and Parkinson's disease(PD) |
|  | **INTRODUCTION** |  |  |  |
| 2 | **Background** | Explain the scientific background and rationale for the reported study. What is the exposure? Is a potential causal relationship between exposure and outcome plausible? Justify why MR is a helpful method to address the study question | 1 | PD is a neurodegenerative disorder, primarily characterized by motor impairments. Vitamin D has several regulatory functions in nerve cell survival and gene expression via its receptors. Although research has shown that vitamin D deficiency is prevalent among PD patients, the causal link to PD risk remains unclear. |
| 3 | **Objectives** | State specific objectives clearly, including pre-specified causal hypotheses (if any). State that MR is a method that, under specific assumptions, intends to estimate causal effects | 1 | This study aims to investigate the causal relationship between vitamin D and PD using a bidirectional two-sample Mendelian Randomization (MR) analysis method. |
|  | **METHODS** |  | 2~4 |  |
| 4 | **Study design and data sources** | Present key elements of the study design early in the article. Consider including a table listing sources of data for all phases of the study. For each data source contributing to the analysis, describe the following: | 2~4 |  |
|  | a) | Setting: Describe the study design and the underlying population, if possible. Describe the setting, locations, and relevant dates, including periods of recruitment, exposure, follow-up, and data collection, when available. | 2~4 | This study employed a bidirectional two-sample MR analysis approach, with the aim of investigating the causal relationship between vitamin D levels and the risk of PD. The data sources were four major public genome-wide association study (GWAS) datasets, encompassing over 1.2 million individuals of European ancestry. |
|  | b) | Participants: Give the eligibility criteria, and the sources and methods of selection of participants. Report the sample size, and whether any power or sample size calculations were carried out prior to the main analysis | 2~4 | Participants were of European descent and the study included data from three PD cohorts. |
|  | c) | Describe measurement, quality control and selection of genetic variants | 2~4 | In this study, researchers selected statistically significant single nucleotide polymorphisms (SNPs) associated with 25-hydroxyvitamin D (25(OH)D) levels as instrumental variables (IVs), to ensure their independence from known confounding factors. The chosen SNPs were required to meet specific criteria such as a P-value less than 5×10-8, R2 less than 0.001, a genetic distance of 10,000 KB, a minor allele frequency greater than 0.01, and an F-statistic greater than 10. |
|  | d) | For each exposure, outcome, and other relevant variables, describe methods of assessment and diagnostic criteria for diseases | 2~4 | It was mentioned that the analysis was conducted using genome-wide association study data, employing inverse-variance weighted (IVW) method and various other MR analytical techniques to assess the relationship between the aggregated SNP statistics and PD. The exposure variable was the level of 25(OH)D, with the outcome being the risk of PD. |
|  | e) | Provide details of ethics committee approval and participant informed consent, if relevant | 2~4 | The study received formal approval from the Medical Ethics Committee of the Second Affiliated Hospital of Xinjiang Medical University (Approval No.: 2022K004). |
| 5 | **Assumptions** | Explicitly state the three core IV assumptions for the main analysis (relevance, independence and exclusion restriction) as well assumptions for any additional or sensitivity analysis | 2~4 | The Relevance Assumption states that the chosen genetic IVs, as principal instrumental variable for analysis, must be significantly associated with 25(OH)D levels. The Independence Assumption requires that the IVs are not associated with any known confounders of the risk of PD. According to the Exclusivity Assumption, the IVs affect the risk of PD solely through the levels of 25(OH)D, without any other pathways. |
| 6 | **Statistical methods: main analysis** | Describe statistical methods and statistics used | 2~4 |  |
|  | a) | Describe how quantitative variables were handled in the analyses (i.e., scale, units, model) | 2~4 | In this study, the levels of 25(OH)D and the risk of PD were analyzed using aggregated statistics from GWAS data. |
|  | b) | Describe how genetic variants were handled in the analyses and, if applicable, how their weights were selected | 2~4 | SNPs significantly associated with 25(OH)D levels were selected as IVs, ensuring they were not related to known confounders. SNPs were chosen based on P values, r-squared (R²), genetic distance, minor allele frequency, and the F-statistic. |
|  | c) | Describe the MR estimator (e.g. two-stage least squares, Wald ratio) and related statistics. Detail the included covariates and, in case of two-sample MR, whether the same covariate set was used for adjustment in the two samples | 2~4 | A consistent covariate selection and adjustment strategy was implemented throughout the analysis of distinct PD cohorts. |
|  | d) | Explain how missing data were addressed | 2~4 | The study utilized data from public databases, and the methods for handling missing data were not elaborated upon. |
|  | e) | If applicable, indicate how multiple testing was addressed | 2~4 | In the selection process for genetic instruments, stringent significance thresholds were employed to reduce the risk of false positives. |
| 7 | **Assessment of assumptions** | Describe any methods or prior knowledge used to assess the assumptions or justify their validity | 2~4 | We selected SNPs significantly associated with 25(OH)D levels based on stringent genome-wide significance thresholds (*P* < 5 × 10^−8^).  We consulted established databases, such as the PhenoScanner, to ensure that the IVs were not linked to known PD risk factors and confounders like C-reactive protein, LDL cholesterol, and HDL cholesterol, thereby satisfying the second condition.  The third principle was primarily assumed due to the biological plausibility and prior knowledge of the direct effects of vitamin D on neural function, although alternative pathways cannot be completely ruled out without further studies. |
| 8 | **Sensitivity analyses and additional analyses** | Describe any sensitivity analyses or additional analyses performed (e.g. comparison of effect estimates from different approaches, independent replication, bias analytic techniques, validation of instruments, simulations) | 2~4 | We deployed multiple MR methods, including IVW, weighted median, MR-Egger, weighted mode, and simple mode, to compare effect estimates from different approaches.  Heterogeneity among instrumental SNPs was examined through Cochrane’s Q test. Additionally, MR-PRESSO analysis identified and adjusted for potential pleiotropic outlier effects, which could bias the results.  We performed leave-one-out sensitivity analysis to determine if any single SNP was exerting undue influence on the overall estimate.  To validate the IVs, F-statistics were calculated for each SNP, selecting only those with an F-value greater than 10 to secure the association.  We ensure the generalizability and credibility of our bidirectional two-sample MR approach by replicating it across three separate PD cohorts. |
| 9 | **Software and pre-registration** |  | 2~4 |  |
|  | a) | Name statistical software and package(s), including version and settings used | 2~4 | The statistical software and packages used in the study "No association between genetically predicted Vitamin D levels and Parkinson’s disease" were R version 4.1.2, with the specific R packages mendelianrandomization, MRPRESSO, and TwoSampleMR. |
|  | b) | State whether the study protocol and details were pre-registered (as well as when and where) | 2~4 | This is a secondary analysis based on summary statistics from existing, published studies. The ethical approval and informed consent have been obtained by all original studies. |
|  | **RESULTS** |  |  |  |
| 10 | **Descriptive data** |  | 4~5 |  |
|  | a) | Report the numbers of individuals at each stage of included studies and reasons for exclusion. Consider use of a flow diagram | 4~5 | Our study followed a strict inclusion and exclusion process for the analysis. A total of 1,275,163 European individuals were considered across four published GWAS datasets. 158 SNPs were initially identified as IVs for 25(OH)D levels. After excluding SNPs based on confounding-related associations, 131 SNPs remained for the final analysis. The reasons for exclusion included significant associations with known confounders such as C-reactive protein, LDL cholesterol, and HDL cholesterol. The selection process for IVs and the MR analysis is depicted in a flow diagram (**Figure 1** in the study). 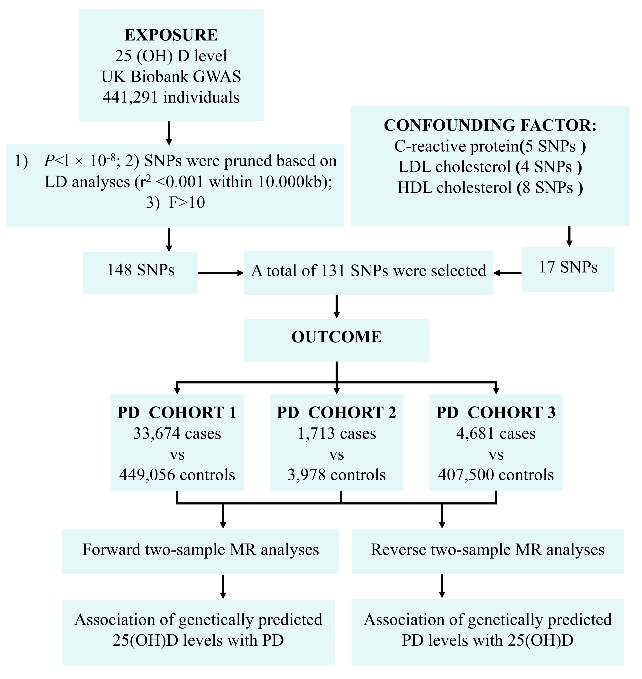 |
|  |  |  |  | **Fig 1. Workflow for selecting IV and MR analysis.** |
|  | b) | Report summary statistics for phenotypic exposure(s), outcome(s), and other relevant variables (e.g. means, SDs, proportions) | 4~5 | SNPs related to 25(OH)D levels were selected based on genome-wide significance (P < 5×10^−8^) from a sample of over 1.2 million individuals. The average F-statistic of these SNPs was 106.64, ranging from 25.33 to 2448.32, indicating strong instrument strength. The MR analysis was performed on data from three PD cohorts, including over 446,000 individuals, both PD cases and controls, with no significant overlap reported between the exposure and outcome datasets. |
|  | c) | If the data sources include meta-analyses of previous studies, provide the assessments of heterogeneity across these studies | 4~5 | Heterogeneity assessments (Cochran's Q test and MR-PRESSO) for the causal estimates across the studies suggested no notable heterogeneity with the exception of PD cohort 2, which showed significant heterogeneity for the relationship between 25(OH)D levels and PD (*P* < 0.05). |
|  | d) | For two-sample MR:  i.  Provide justification of the similarity of the genetic variant-exposure associations between the exposure and outcome samples  ii.  Provide information on the number of individuals who overlap between the exposure and outcome studies | 4~5 | i. The genetic variants used as IVs for 25(OH)D levels were validated to have consistent associations with the exposure in both the outcome and exposure samples.  ii. The datasets from the exposure and outcome cohorts were distinct with no information suggesting a significant number of individuals overlapping between the exposure and outcome studies. |
| 11 | **Main results** |  | 4~5 |  |
|  | a) | Report the associations between genetic variant and exposure, and between genetic variant and outcome, preferably on an interpretable scale | 4~5 | The associations between the selected genetic variants and 25(OH)D levels, as well as between the genetic variants and PD, were analyzed. No significant association was found between the genetically predicted 25(OH)D levels and PD risk using the IVW method (*P*_cohort1_ = 0.365, *P*_cohort2_ = 0.525, *P*_cohort3_ = 0.117) or additional MR methods (weighted median, simple mode, and weighted mode). |
|  | b) | Report MR estimates of the relationship between exposure and outcome, and the measures of uncertainty from the MR analysis, on an interpretable scale, such as odds ratio or relative risk per SD difference | 4~5 | The MR estimates showed no significant relationship between 25(OH)D levels and PD risk. |
|  | c) | If relevant, consider translating estimates of relative risk into absolute risk for a meaningful time period | 4~5 | Given the non-significant MR estimates, converting the relative risks into absolute risks was not applicable. |
|  | d) | Consider plots to visualize results (e.g. forest plot, scatterplot of associations between genetic variants and outcome versus between genetic variants and exposure) | 4~5 | The MR analysis results are visualized using plots (e.g., forest plots and scatterplots) to illustrate the lack of association between genetic variants related to 25(OH)D levels and the risk of PD (Figures 2 and 3). Forest plots show the individual SNP effect sizes and the overall estimate, while scatterplots demonstrate the associations between genetic variants and the risk of PD versus the genetic variants and 25(OH)D levels. |
| 12 | **Assessment of assumptions** |  | 4~5 |  |
|  | a) | Report the assessment of the validity of the assumptions |  | This study utilized a bidirectional two-sample MR approach to investigate the potential causal link between vitamin D and PD. Three critical assumptions underpin the efficacy of the IVs: firstly, the genetic variants used as IVs exhibit a significant correlation with the exposure factor (25(OH)D levels); secondly, these genetic variants should not be correlated with any confounding variables; thirdly, the genetic variants must affect the outcome (PD) solely through the exposure factor, without any alternative pathways. In the course of the analysis, SNPs related to established confounding factors such as C-reactive protein, LDL cholesterol, and HDL cholesterol were assessed for correlation and consequently excluded. This process enhanced the IVs' efficacy and the accuracy of the causal inference. |
|  | b) | Report any additional statistics (e.g., assessments of heterogeneity across genetic variants, such as *I^2^*, Q statistic or E-value) | 4~5 | The GWAS data utilized in this research were derived from four major published datasets involving over 1.2 million Europeans, which facilitated the elucidation of the genetic correlation between Vitamin D levels and PD risk. Data from three PD cohorts did not exhibit a significant association between 25(OH)D levels and PD risk, according to the analyses conducted using the IVW method (*P*_cohort1_ = 0.365, *P*_cohort2_ = 0.525, *P*_cohort3_ = 0.117). To evaluate heterogeneity among different genetic variants, this study made use of Cochrane's Q test. Heterogeneity was coded in PD cohort 2 (*P* < 0.05), with MR-Egger regression and one-to-one analysis revealing an absence of significant heterogeneity or pleiotropy (refer to **Table 1** and **Figure S1**). |
| 13 | **Sensitivity analyses and additional analyses** |  | 4~5 |  |
|  | a) | Report any sensitivity analyses to assess the robustness of the main results to violations of the assumptions | 4~5 | Sensitivity analysis results were consistent with the primary analysis, indicating no significant causal relationship between genetically predicted vitamin D levels and PD. Heterogeneity indicated by Cochrane's Q test was *P*_cohort2_ = 0.019, with no significant pleiotropy detected by MR-Egger regression (*P* > 0.05). |
|  | b) | Report results from other sensitivity analyses or additional analyses | 4~5 | Sensitivity analysis results were consistent with the primary analysis, indicating no significant causal relationship between genetically predicted vitamin D levels and PD. Heterogeneity indicated by Cochrane's Q test was P_cohort2_=0.019, with no significant pleiotropy detected by MR-Egger regression (*P* > 0.05). |
|  | c) | Report any assessment of direction of causal relationship (e.g., bidirectional MR) | 4~5 | Our bidirectional MR analysis evaluated the causal relationship between vitamin D levels and PD in both directions. Forward MR showed no causal effect of vitamin D levels on the risk of PD, whereas the reverse MR indicated no evidence that PD risk causally decreases vitamin D levels. |
|  | d) | When relevant, report and compare with estimates from non-MR analyses | 4~5 | Comparing our MR estimates with results from observational studies revealed that neither method supports a significant association between vitamin D levels and the risk of PD. |
|  | e) | Consider additional plots to visualize results (e.g., leave-one-out analyses) | 4~5 | Additional figures, such as scatter plots and funnel plots correlating SNPs with vitamin D levels and PD risk, were included to assess pleiotropy. Leave-one-out analysis was also conducted to ensure no single SNP disproportionately affected the overall MR estimates. |
|  | **DISCUSSION** |  |  |  |
| 14 | **Key results** | Summarize key results with reference to study objectives | 6~7 | Our bidirectional MR analysis did not reveal a significant causal association between genetically predicted serum Vitamin D levels and PD, aligning with our study's objective to clarify this potential relationship. |
| 15 | **Limitations** | Discuss limitations of the study, taking into account the validity of the IV assumptions, other sources of potential bias, and imprecision. Discuss both direction and magnitude of any potential bias and any efforts to address them | 6~7 | Firstly, although this study selected SNPs significantly associated with 25(OH)D levels as an IV to ensure it was uncorrelated with known confounders, the validity of the IV still remains a potential limitation. The selection of SNPs was based on their association with 25(OH)D levels, but it cannot be guaranteed that these SNPs do not have a direct effect on PD risk, nor can the presence of undetected confounding factors be ruled out, which may introduce certain biases.  Secondly, although we utilized five different MR methods to assess the heterogeneity of SNPs and conducted outlier correction with MR-PRESSO analysis, these methods cannot completely eliminate all possible pleiotropic effects. Furthermore, Cochran's Q test for unidirectional and bidirectional MR exhibited some level of heterogeneity in certain cohorts, although MR-Egger regression test and leave-one-out analysis did not show significant heterogeneity or pleiotropy.  Thirdly,The study's sample was also limited to individuals of European ancestry, therefore, the findings may not apply to groups from other racial and ethnic backgrounds. The potential effects of population stratification would need to be further investigated by including samples from a wider range of ethnicities. |
| 16 | **Interpretation** |  | 6~7 |  |
|  | a) | Meaning: Give a cautious overall interpretation of results in the context of their limitations and in comparison with other studies | 6~7 | Given the limitations inherent to this research design and the null findings in comparison to other studies, we cautiously interpret that the current evidence suggests no substantial genetic association between vitamin D levels and PD risk. While vitamin D deficiency is common in PD patients, our bidirectional MR analysis across three cohorts did not demonstrate a significant causal relationship. These results align with previous observational studies that also showed no direct correlation. The study design is robust, utilizing a large dataset and a bidirectional approach to strengthen the reliability and validity of the findings. |
|  | b) | Mechanism: Discuss underlying biological mechanisms that could drive a potential causal relationship between the investigated exposure and the outcome, and whether the gene-environment equivalence assumption is reasonable. Use causal language carefully, clarifying that IV estimates may provide causal effects only under certain assumptions | 6~7 | The biological plausibility of vitamin D playing a role in PD is underscored by its involvement in neuronal survival and gene expression. The neuroprotective properties could stem from its regulatory functions, preventing cytotoxicity and fostering neural growth. However, given the complexity of PD etiology and the multitude of factors involved, the gene-environment equivalence assumption for MR analyses is still speculative. Careful use of causal language is required as the estimated effects derived from IVs are contingent upon the validity of the MR assumptions. In our study, the lack of association highlights that while genetically predicted serum vitamin D levels might not causally influence PD risk, it does not dismiss other potential pathways and mechanisms through which vitamin D may exhibit effects on neuronal health. |
|  | c) | Clinical relevance: Discuss whether the results have clinical or public policy relevance, and to what extent they inform effect sizes of possible interventions | 6~7 | Although the results indicate no genetic causal link between vitamin D levels and PD risk, they do contribute to the understanding of PD pathophysiology and have implications for clinical and public health strategies. Ensuring adequate vitamin D status remains essential for overall health and may still be beneficial for non-genetic aspects of PD. However, the genetic component of vitamin D as a preventive or therapeutic measure for PD appears to be minimal based on our findings. Further research including different ethnicities and longer-term vitamin D status measures are required to evaluate whether these results are applicable more broadly. If similar findings are consistently observed across diverse populations, this would have significant implications for the focus and direction of future PD research and interventions. |
| 17 | **Generalizability** | Discuss the generalizability of the study results (a) to other populations, (b) across other exposure periods/timings, and (c) across other levels of exposure | 6~7 | The study sample comprised individuals of European descent, therefore our results may not be directly applicable to other ethnic groups due to genetic and environmental diversity. Although genetic associations can be consistent across different populations, allele frequencies and the presence of confounding factors may vary. To enhance generalizability, we recommend replication of the study across diverse populations. The association between vitamin D levels and PD was analyzed at genetically predicted levels of vitamin D. This approach does not fully assess all levels of exposure, such as high-dose vitamin D supplementation or severe deficiencies which may have different effects on PD risk. Subsequent observational studies or randomized trials with varying doses could provide additional insights into dose-response relationships. |
|  | **OTHER INFORMATION** |  |  |  |
| 18 | **Funding** | Describe sources of funding and the role of funders in the present study and, if applicable, sources of funding for the databases and original study or studies on which the present study is based | Information in submission | This research was supported by the Autonomous Region Key Research and Development Project (Grant No. 2023B03003), the Tian-Shan Talent Program (Grant No. 2022TSYCLJ0066), the National Natural Science Foundation of China (Grant No. 82371258), and the Central Guiding Local Science and Technology Development Special Fund Project (Grant No. ZYYD2022C17). |
| 19 | **Data and data sharing** | Provide the data used to perform all analyses or report where and how the data can be accessed, and reference these sources in the article. Provide the statistical code needed to reproduce the results in the article, or report whether the code is publicly accessible and if so, where | 3~4 | To perform all analyses or report on this study, we have utilized data from publicly accessible genome-wide association studies (GWAS). The 25(OH)D GWAS data was derived from a study by Manousaki et al. conducted using the UK Biobank cohort. This dataset involves SNP data from 401,460 individuals of European ancestry. For PD analysis, data were sourced from three PD outcome cohorts' GWAS data, which include:  1. GWAS ID "ieu-b-7" from the International PD Genomics Consortium (IPDGC), incorporating three previously reported studies, 13 new datasets, and proxy case data from the UK Biobank, encompassing 33,674 cases and 449,056 controls.  2. GWAS ID "ieu-a-812", which includes 1,713 Caucasian patients and 3,978 healthy controls.  3. GWAS ID "finn-b-G6_PARKINSON" from the FinnGen Consortium, used for MR analysis with 4,681 cases and 407,500 controls.  These data are available for further analysis and can be accessed through the respective public GWAS databases. For researchers interested in replicating or extending the analysis, the statistical code and methodology used are available upon request by contacting the corresponding author via email at *yangxinling2014@163.com*. |
| 20 | **Conflicts of Interest** | All authors should declare all potential conflicts of interest | Information in submission | The authors declare no competing interests. |

This checklist is copyrighted by the Equator Network under the Creative Commons Attribution 3.0 Unported (CC BY 3.0) license.

1. Skrivankova VW, Richmond RC, Woolf BAR, Yarmolinsky J, Davies NM, Swanson SA, et al. Strengthening the Reporting of Observational Studies in Epidemiology using Mendelian Randomization (STROBE-MR) Statement. JAMA. 2021;under review.

2. Skrivankova VW, Richmond RC, Woolf BAR, Davies NM, Swanson SA, VanderWeele TJ, et al. Strengthening the Reporting of Observational Studies in Epidemiology using Mendelian Randomisation (STROBE-MR): Explanation and Elaboration. BMJ. 2021;375:n2233.
