# Supplementary material for: No association between genetically predicted vitamin D levels and Parkinson’s disease
Source: PLoS One. 2024 Nov 15;19(11):e0313631. doi: 10.1371/journal.pone.0313631 (PMC11567546; doi:10.1371/journal.pone.0313631)
Supplement: S1 Table — (DOCX) [file pone.0313631.s002.docx]

**Supplementary Material**

**No association between genetically predicted Vitamin D levels and Parkinson's disease**

**Tables S1. SNPs associated with 25(OH)D**

| **SNP** | **Effect_allele** | **Other_allele** | **Beta** | **SE** | **R^2^** | **F** |
| --- | --- | --- | --- | --- | --- | --- |
| rs6698680 | G | A | -0.0119 | 0.0019 | 0.0001 | 37.53 |
| rs3750296 | C | G | -0.0208 | 0.0020 | 0.0002 | 103.94 |
| rs7519574 | A | G | 0.0170 | 0.0025 | 0.0001 | 44.89 |
| rs72665698 | G | T | 0.0140 | 0.0023 | 0.0001 | 35.98 |
| rs512083 | C | T | 0.0100 | 0.0019 | 0.0001 | 26.24 |
| rs2934744 | C | A | 0.0224 | 0.0021 | 0.0003 | 111.80 |
| rs11209952 | T | C | -0.0105 | 0.0020 | 0.0001 | 28.40 |
| rs12123821 | T | C | 0.0745 | 0.0046 | 0.0006 | 264.05 |
| rs61815559 | T | A | 0.0840 | 0.0055 | 0.0005 | 230.70 |
| rs10908469 | C | A | 0.0160 | 0.0022 | 0.0001 | 53.82 |
| rs61747728 | T | C | 0.0317 | 0.0051 | 0.0001 | 38.92 |
| rs2647463 | C | T | -0.0108 | 0.0020 | 0.0001 | 28.00 |
| rs867772 | G | A | -0.0138 | 0.0021 | 0.0001 | 43.80 |
| rs4635554 | G | T | -0.0127 | 0.0021 | 0.0001 | 38.18 |
| rs1800440 | C | T | -0.0140 | 0.0025 | 0.0001 | 31.44 |
| rs6741951 | A | G | -0.0115 | 0.0022 | 0.0001 | 28.56 |
| rs55886116 | T | G | 0.0145 | 0.0025 | 0.0001 | 34.14 |
| rs6724965 | G | A | -0.0165 | 0.0026 | 0.0001 | 41.33 |
| rs7569755 | A | G | 0.0139 | 0.0021 | 0.0001 | 42.25 |
| rs13030535 | T | C | 0.0102 | 0.0020 | 0.0001 | 26.38 |
| rs11885466 | T | C | -0.0188 | 0.0037 | 0.0001 | 25.93 |
| rs2012736 | A | C | -0.0456 | 0.0036 | 0.0004 | 160.78 |
| rs4603973 | G | T | -0.0123 | 0.0023 | 0.0001 | 29.64 |
| rs76183418 | C | T | 0.0131 | 0.0026 | 0.0001 | 25.43 |
| rs34293138 | C | T | -0.0124 | 0.0021 | 0.0001 | 34.72 |
| rs1972994 | T | A | -0.0175 | 0.0020 | 0.0002 | 73.95 |
| rs1909585 | T | C | 0.0105 | 0.0021 | 0.0001 | 25.70 |
| rs6438900 | G | C | 0.0136 | 0.0022 | 0.0001 | 37.41 |
| rs6773343 | T | C | 0.0127 | 0.0022 | 0.0001 | 34.11 |
| rs112736716 | C | T | 0.0106 | 0.0021 | 0.0001 | 25.96 |
| rs1542926 | C | T | 0.0164 | 0.0031 | 0.0001 | 28.44 |
| rs80237449 | G | A | 0.0178 | 0.0034 | 0.0001 | 27.56 |
| rs78649910 | A | T | -0.0183 | 0.0031 | 0.0001 | 34.48 |
| rs4364259 | A | G | 0.0158 | 0.0024 | 0.0001 | 42.55 |
| **SNP** | **Effect_allele** | **Other_allele** | **Beta** | **SE** | **R^2^** | **F** |
| rs4616820 | T | C | -0.0110 | 0.0019 | 0.0001 | 32.18 |
| rs12500806 | T | C | 0.0124 | 0.0023 | 0.0001 | 27.68 |
| rs7699711 | T | G | -0.0286 | 0.0019 | 0.0005 | 215.93 |
| rs4645189 | T | C | 0.0234 | 0.0026 | 0.0002 | 80.58 |
| rs4694423 | A | C | -0.0973 | 0.0020 | 0.0055 | 2448.33 |
| rs71601787 | A | G | 0.0422 | 0.0021 | 0.0009 | 414.78 |
| rs7657132 | G | A | -0.0149 | 0.0021 | 0.0001 | 51.20 |
| rs58073039 | G | A | -0.0141 | 0.0021 | 0.0001 | 44.82 |
| rs116472025 | A | G | 0.0373 | 0.0060 | 0.0001 | 38.07 |
| rs27774 | A | G | -0.0121 | 0.0021 | 0.0001 | 31.88 |
| rs7718395 | G | C | 0.0126 | 0.0021 | 0.0001 | 36.32 |
| rs466360 | A | G | -0.0114 | 0.0020 | 0.0001 | 32.61 |
| rs7724488 | G | A | 0.0117 | 0.0020 | 0.0001 | 34.57 |
| rs12196316 | C | T | -0.0124 | 0.0021 | 0.0001 | 34.00 |
| rs28798705 | G | A | -0.0123 | 0.0023 | 0.0001 | 29.34 |
| rs9476310 | T | C | 0.0111 | 0.0019 | 0.0001 | 32.66 |
| rs35050704 | A | G | 0.0156 | 0.0030 | 0.0001 | 28.00 |
| rs942380 | G | A | 0.0114 | 0.0020 | 0.0001 | 33.31 |
| rs2245133 | C | T | -0.0209 | 0.0026 | 0.0001 | 63.48 |
| rs111529171 | C | G | -0.0155 | 0.0024 | 0.0001 | 42.74 |
| rs62461553 | A | C | 0.0106 | 0.0020 | 0.0001 | 28.14 |
| rs112989220 | A | G | 0.0100 | 0.0020 | 0.0001 | 25.40 |
| rs75741381 | G | C | -0.0163 | 0.0027 | 0.0001 | 35.79 |
| rs1011468 | A | G | -0.0138 | 0.0019 | 0.0001 | 50.25 |
| rs2395839 | G | A | 0.0099 | 0.0020 | 0.0001 | 25.87 |
| rs10231102 | A | G | -0.0110 | 0.0020 | 0.0001 | 30.71 |
| rs804280 | A | C | 0.0130 | 0.0020 | 0.0001 | 43.41 |
| rs34726834 | T | C | 0.0138 | 0.0022 | 0.0001 | 38.12 |
| rs4738679 | A | G | 0.0107 | 0.0021 | 0.0001 | 26.81 |
| rs10101205 | C | T | 0.0138 | 0.0027 | 0.0001 | 26.40 |
| rs13278404 | G | C | 0.0146 | 0.0028 | 0.0001 | 26.61 |
| rs10758321 | A | G | 0.0101 | 0.0020 | 0.0001 | 25.85 |
| rs13284054 | C | T | 0.0173 | 0.0030 | 0.0001 | 33.18 |
| rs12554549 | T | C | 0.0209 | 0.0040 | 0.0001 | 27.34 |
| rs10818769 | G | C | -0.0170 | 0.0029 | 0.0001 | 34.97 |
| rs9423639 | T | C | -0.0120 | 0.0022 | 0.0001 | 30.15 |
| rs4553272 | T | C | -0.0104 | 0.0020 | 0.0001 | 28.00 |
| rs10887718 | T | C | -0.0125 | 0.0019 | 0.0001 | 41.10 |
| rs2607863 | C | T | -0.0246 | 0.0043 | 0.0001 | 32.79 |
| rs11602347 | G | C | 0.0101 | 0.0020 | 0.0001 | 25.66 |
| rs138072379 | T | C | 0.0426 | 0.0069 | 0.0001 | 38.15 |
| rs182244780 | A | G | -0.3353 | 0.0085 | 0.0035 | 1550.47 |
| rs55665837 | T | C | -0.0599 | 0.0020 | 0.0020 | 890.08 |
| **SNP** | **Effect_allele** | **Other_allele** | **Beta** | **SE** | **R^2^** | **F** |
| rs550369460 | T | C | 0.0647 | 0.0105 | 0.0001 | 37.82 |
| rs146128209 | G | A | -0.0470 | 0.0038 | 0.0004 | 155.60 |
| rs11500197 | A | G | -0.0122 | 0.0022 | 0.0001 | 30.15 |
| rs4930354 | C | T | 0.0118 | 0.0020 | 0.0001 | 36.14 |
| rs144685272 | A | G | -0.0306 | 0.0056 | 0.0001 | 29.94 |
| rs62645169 | T | G | 0.0201 | 0.0024 | 0.0002 | 71.39 |
| rs12803256 | G | A | 0.1003 | 0.0023 | 0.0042 | 1861.96 |
| rs10899104 | G | T | 0.0128 | 0.0025 | 0.0001 | 25.86 |
| rs2847500 | A | G | -0.0211 | 0.0029 | 0.0001 | 51.33 |
| rs12317268 | G | A | -0.0185 | 0.0027 | 0.0001 | 46.50 |
| rs10771090 | G | A | -0.0107 | 0.0020 | 0.0001 | 30.00 |
| rs9668081 | T | C | 0.0116 | 0.0020 | 0.0001 | 34.05 |
| rs784887 | G | T | -0.0138 | 0.0026 | 0.0001 | 29.02 |
| rs59789656 | T | C | -0.0125 | 0.0023 | 0.0001 | 29.49 |
| rs10859995 | C | T | -0.0394 | 0.0020 | 0.0009 | 399.51 |
| rs75936148 | A | G | -0.0172 | 0.0033 | 0.0001 | 26.58 |
| rs11060406 | T | C | -0.0273 | 0.0053 | 0.0001 | 26.95 |
| rs9569235 | C | A | -0.0127 | 0.0022 | 0.0001 | 33.80 |
| rs7981402 | A | G | 0.0104 | 0.0021 | 0.0001 | 25.85 |
| rs1191520 | C | T | -0.0108 | 0.0021 | 0.0001 | 27.51 |
| rs8018720 | C | G | -0.0319 | 0.0025 | 0.0004 | 157.47 |
| rs72680101 | A | G | -0.0276 | 0.0051 | 0.0001 | 28.96 |
| rs745797 | A | G | -0.0149 | 0.0029 | 0.0001 | 25.92 |
| rs7148857 | G | C | -0.0133 | 0.0021 | 0.0001 | 39.15 |
| rs17651741 | A | G | -0.0134 | 0.0025 | 0.0001 | 29.75 |
| rs174418 | C | T | 0.0219 | 0.0020 | 0.0003 | 121.49 |
| rs4267257 | G | A | -0.0144 | 0.0026 | 0.0001 | 30.99 |
| rs7178572 | G | A | -0.0145 | 0.0022 | 0.0001 | 45.38 |
| rs34560261 | T | C | 0.0136 | 0.0027 | 0.0001 | 26.01 |
| rs8063706 | T | A | 0.0130 | 0.0022 | 0.0001 | 34.81 |
| rs77924615 | A | G | -0.0158 | 0.0025 | 0.0001 | 41.08 |
| rs35733741 | A | G | 0.0117 | 0.0020 | 0.0001 | 34.72 |
| rs77194050 | G | A | 0.0232 | 0.0044 | 0.0001 | 27.13 |
| rs9926530 | G | T | 0.0222 | 0.0043 | 0.0001 | 26.21 |
| rs11542462 | A | G | -0.0218 | 0.0029 | 0.0001 | 57.50 |
| rs72631431 | T | C | 0.0107 | 0.0021 | 0.0001 | 25.33 |
| rs2909218 | T | C | 0.0169 | 0.0024 | 0.0001 | 48.81 |
| rs8091117 | A | C | -0.0241 | 0.0039 | 0.0001 | 37.27 |
| rs80204526 | A | C | -0.0482 | 0.0094 | 0.0001 | 26.20 |
| rs2037511 | A | G | 0.0160 | 0.0026 | 0.0001 | 37.47 |
| rs73015021 | G | A | 0.0230 | 0.0030 | 0.0001 | 59.63 |
| rs424132 | A | T | -0.0117 | 0.0020 | 0.0001 | 33.10 |
| rs3814995 | T | C | -0.0147 | 0.0021 | 0.0001 | 48.80 |
| **SNP** | **Effect_allele** | **Other_allele** | **Beta** | **SE** | **R^2^** | **F** |
| rs5112 | G | C | -0.0149 | 0.0021 | 0.0001 | 49.37 |
| rs112285002 | T | C | 0.0603 | 0.0027 | 0.0011 | 498.75 |
| rs1048328 | A | G | 0.0283 | 0.0036 | 0.0001 | 61.64 |
| rs67127431 | A | C | 0.0111 | 0.0022 | 0.0001 | 26.31 |
| rs11606 | G | C | 0.0109 | 0.0020 | 0.0001 | 29.70 |
| rs56257628 | A | C | -0.0160 | 0.0030 | 0.0001 | 27.93 |
| rs6127099 | T | A | -0.0368 | 0.0022 | 0.0006 | 274.98 |
| rs2762938 | A | G | 0.0133 | 0.0020 | 0.0001 | 43.33 |
| rs2229742 | C | G | -0.0257 | 0.0032 | 0.0001 | 65.10 |
| rs2074735 | C | G | 0.0273 | 0.0040 | 0.0001 | 47.16 |
| rs9616951 | T | C | -0.0113 | 0.0021 | 0.0001 | 28.88 |

SE, Standard Error; SNPs, single nucleotide polymorphisms; 25(OH)D, 25-hydroxyvitamin D.
