# Supplementary figures and images for: No association between genetically predicted vitamin D levels and Parkinson’s disease
Source: PLoS One. 2024 Nov 15;19(11):e0313631. doi: 10.1371/journal.pone.0313631 (PMC11567546; doi:10.1371/journal.pone.0313631)

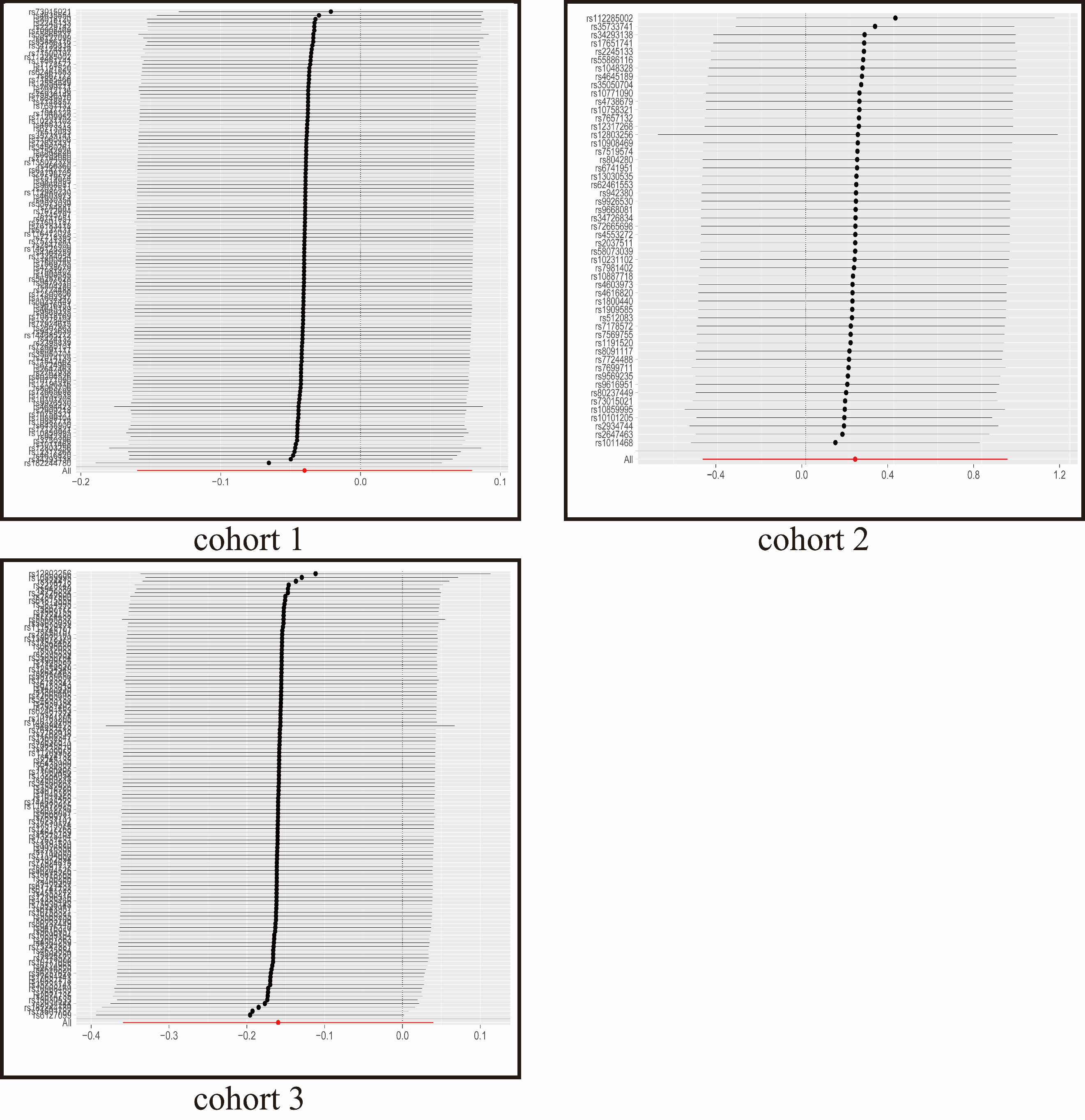


**Fig S1. Leave-one-out analyses for each SNP-25(OH)D association**

Supplement: S1 Fig — (DOCX) [file pone.0313631.s003.docx]

**
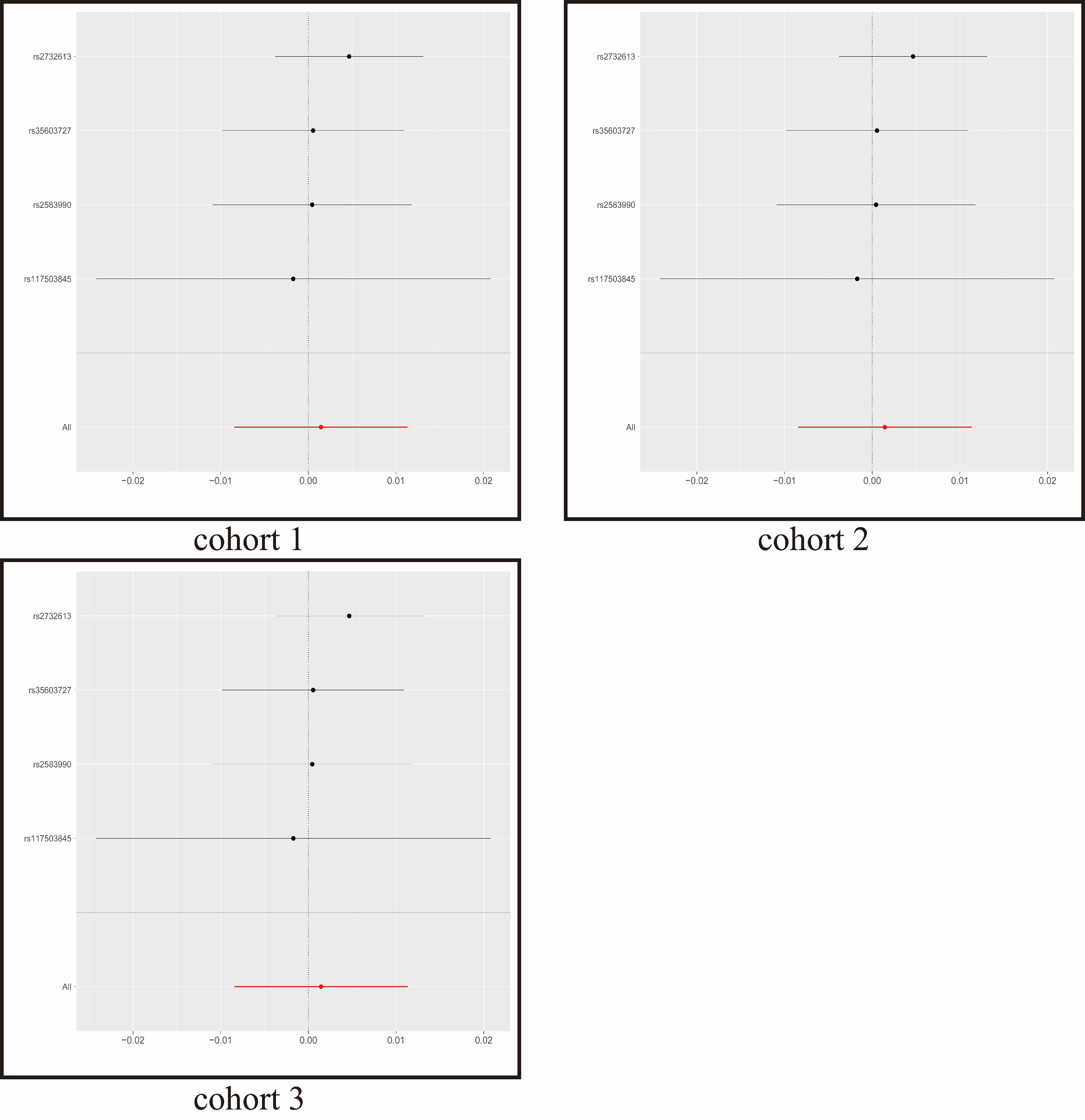
**

**Fig S2. Leave-one-out analyses for each SNP-PD association**

Supplement: S2 Fig — (DOCX) [file pone.0313631.s004.docx]
